# USP18 – a multifunctional component in the interferon response

**DOI:** 10.1042/BSR20180250

**Published:** 2018-11-16

**Authors:** Anja Basters, Klaus-Peter Knobeloch, Günter Fritz

**Affiliations:** 1 Friedrich Miescher Institute for Biomedical Research, Quantitative Biology, Basel, Switzerland; 2 Faculty of Medicine, Institute of Neuropathology, University of Freiburg, Freiburg, Germany; 3 Institute of Microbiology, University of Hohenheim, Stuttgart, Germany

**Keywords:** ISG15, interferons, protease, ubiquitin like modifier proteins, USP18

## Abstract

Ubiquitin-specific proteases (USPs) represent the largest family of deubiquitinating enzymes (DUB). These proteases cleave the isopeptide bond between ubiquitin and a lysine residue of a ubiquitin-modified protein. USP18 is a special member of the USP family as it only deconjugates the ubiquitin-like protein ISG15 (interferon-stimulated gene (ISG) 15) from target proteins but is not active towards ubiquitin. Independent of its protease activity, USP18 functions as a major negative regulator of the type I interferon response showing that USP18 is – at least – a bifunctional protein. In this review, we summarise our current knowledge of protease-dependent and -independent functions of USP18 and discuss the structural basis of its dual activity.

## Introduction

**Figure 1:**
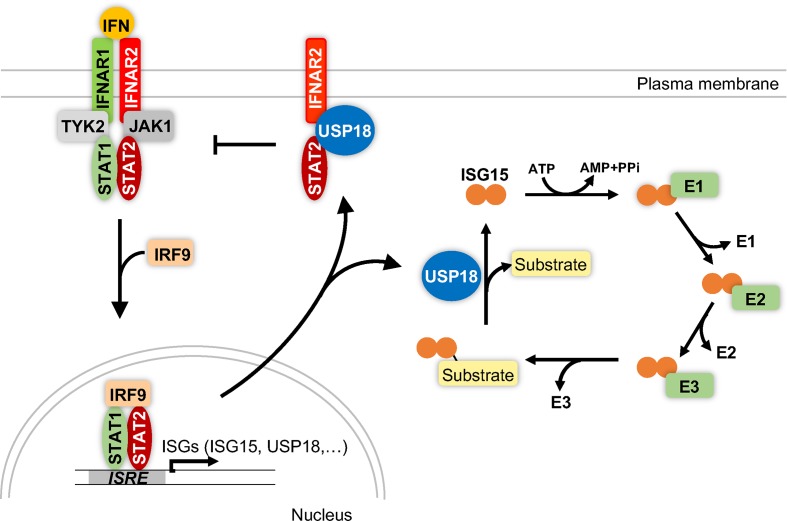
Enzymatic and non-enzymatic functions of USP18 Type I interferons (IFN) bind to a dimeric receptor (IFNAR1 and IFNAR2) on the cell surface to activate the intracellular kinases TYK2 and JAK1. These kinases recruit and phosphorylate the transcription factors STAT1 and STAT2 which subsequently bind the transcription factor IRF9 and translocate into the nucleus. The trimeric complex binds to the ISRE element in promoters of interferon-stimulated genes (ISGs) and induces the expression of ISGs including USP18, ISG15 and the E1, E2 and E3 enzymes that catalyse ISGylation of substrate proteins. USP18 deISGylates target proteins by cleaving the isopeptide bond between the C-terminus of ISG15 and a lysine residue of the substrate protein. USP18 also interacts with IFNAR2 and STAT2 to block type I interferon signalling in a protease-independent manner. Abbreviations: IRF9, IFN-regulatory factor 9, JAK1, Janus activated kinase 1; STAT, signal transducer and activator of transcription; TYK2, tyrosine kinase 1.

The Ubiquitin-specific protease (USP) 18 (USP18, also known as UBP43) was first cloned from mice [1] and later also from human cells [2]. On the basis of its sequence, the protein was assigned to the family of USPs. The expression of USP18 is strongly induced by type I and type III interferons [2–5], by the Toll-like receptor (TLR) agonists LPS [6–8] and polyI:C [6] (synthetic analogy of dsRNA) and by TNFα [7]. In line with this, USP18 RNA and protein levels in cells are increased after viral or bacterial infection [9–12]. USP18 specifically deconjugates the ubiquitin-like protein (Ubl) *ISG15* (interferon-stimulated gene 15) from target proteins [13–15]. ISG15 comprises two ubiquitin-like domains that are connected by a flexible linker [16]. Analogous to the post-translational modification with ubiquitin, ISG15 is conjugated to target proteins by the consecutive action of an E1 activating enzyme (Ube1l) [17], an E2 conjugating enzyme (UbcH8) [18] and an E3 ligase (Herc5 in humans or Herc6 in mice) [19–21]. Besides being an active enzyme, USP18 negatively regulates type I interferon signalling independent of its protease activity [22] (Figure 1).

## USP18 – the major ISG15 isopeptidase

The members of the USP family share a common molecular architecture and comprise three domains which are called finger, thumb and palm domain [23]. USPs are cysteine proteases with a catalytic triad composed of a cysteine, a histidine and an aspartate or asparagine residues [23]. This catalytic triad is located at the interface between the palm and the thumb domain and is required to cleave the isopeptide bond between ubiquitin or an Ubl and the lysine residue of the target protein [24]. USPs are regarded as rather unspecific enzymes as most USPs cleave ubiquitin chains independent of the respective linkage type [23]. In addition, USP2, USP5, USP13, USP14 and USP21 have been shown to recognise both ubiquitin and ISG15 [25,26]. In contrast with those promiscuous members of the USP family, USP18 is highly specific for ISG15 and does not show cross-reactivity towards ubiquitin [13–15]. Moreover, USP18 represents the major ISG15 deconjugating enzyme *in vivo*: mice that express a catalytic inactive version of USP18 (USP18-C61A, the catalytic cysteine has been replaced by alanine) show increased levels of ISGylation after stimulation with IFNβ in a wide variety of organs and cell types such as lung, lymph nodes, spleen, thymus, liver and bone-marrow derived macrophages [27]. Thus, loss of USP18 catalytic activity cannot be compensated by any other ISG15 isopeptidase. In addition, USP18-mediated deISGylation *in vitro* is approximately 40-fold faster than deISGylation by the cross-reactive deubiquitinating enzymes (DUB) USP21 [15] raising the question whether deISGylation by Ub/ISG15 cross-reactive DUBs is relevant *in vivo*.

Despite enhanced ISGylation, mice homozygous for USP18-C61A (USP18^C61A/C61A^) are healthy and display a normal lifespan [27]. In addition, USP18^C61A/C61A^ mice show increased resistance to infection with vaccinia and influenza B virus as well as against Coxsackie b virus induced myocarditis highlighting the importance of the protease function of USP18 in viral infections [27,28]. For influenza B virus, it has been shown that the increased resistance observed for USP18^C61A/C61A^ mice can be reversed if mice additionally lack ISG15 [27]. Thus, inhibiting USP18 catalytic activity might represent a novel strategy to counteract viral infection. The importance of ISG15 in defeating viral infections is underlined by the fact that several viruses counteract ISGylation and express proteins that either bind ISG15 (Influenza B) [29,30] or proteases that deconjugate ISG15 from proteins (foot-and-mouth disease virus [31], Crimean Congo Hemorrhagic Fever Virus [32,33], SARS-corona virus [34], MERS-corona virus [34]).

## Structure of USP18

**Figure 2:**
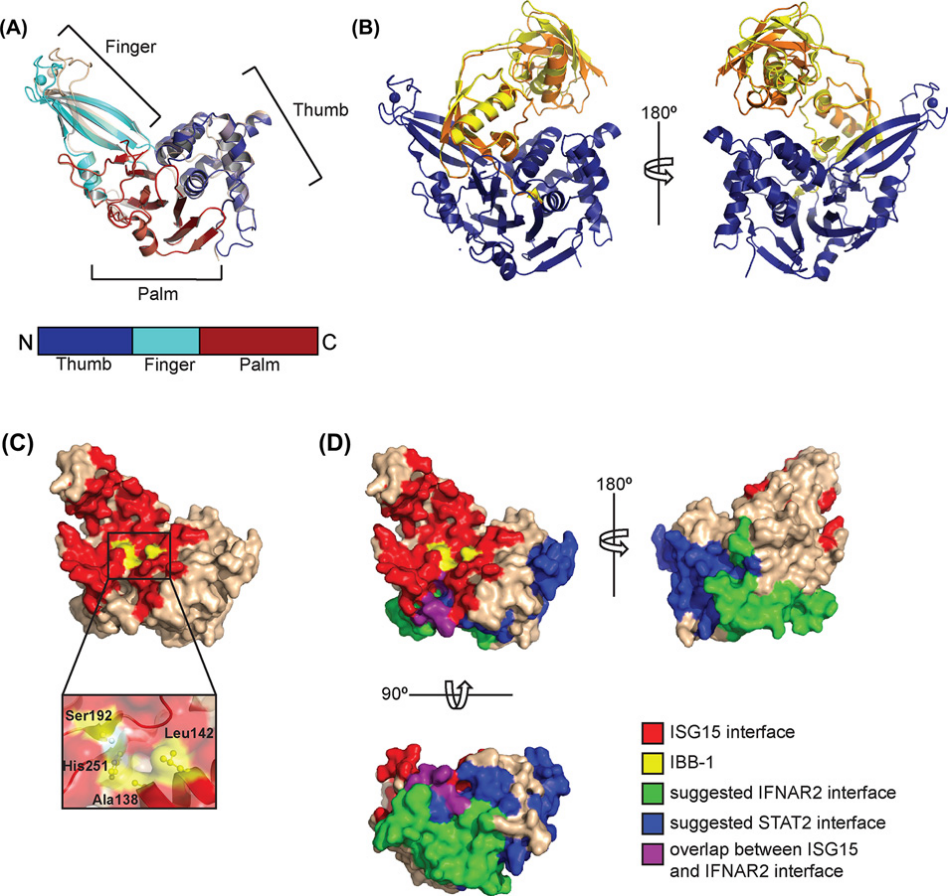
Structure of free and ISG15-bound USP18 (**A**) Overall structure of USP18 (pdb code 5cht) showing the three-domain architecture with finger, thumb and palm domains. The finger domain co-ordinates a Zn ion shown as sphere. Superposition of the two chains present in the asymmetric unit revealed that the enzyme had crystallised in two conformations that mainly differ in the orientation of the finger domain. (**B**) Structure of the USP18–ISG15 complex (pdb code 5chv). Only the C-terminal Ubl domain of ISG15 makes extensive contacts with all three domains of USP18 (blue) and the C-terminal tail of ISG15 lies in the cleft between the palm and the thumb domains where it reaches the catalytic triad. The two ISG15 molecules present in the asymmetric unit differ in the relative orientation of N- and C-terminal Ubl domains whereas the two USP18 molecules are virtually identical. The ISG15 molecules are shown in yellow and orange, USP18 in blue. (**C**) Surface representation of USP18 in the active conformation (ISG15-bound). The surface area with which USP18 recognises ISG15 was calculated by the Eppic server (software version 3.0.4) [64] and is shown in red. The residues that form ISG15-binding box1 (IBB-1) are depicted in yellow and shown as a close-up view below. (**D**) The suggested surface areas with which USP18 binds to STAT2 and IFNAR2 are depicted in blue and green, respectively. These surface areas were defined using deletion constructs of human USP18 [22,58] and the respective residues of mouse USP18 are shown. For IFNAR2 an additional interface was mapped in the N-terminus of USP18 [58] which is not present in the structure. A small surface area on USP18 interacts with ISG15 and was also suggested to bind to IFNAR2. This area is shown in purple.

While the role of USP18 isopeptidase activity *in vivo* has been the topic of numerous studies, the crystal structure of USP18 was described only recently [35,36]. USP18 adopts a 3D architecture similar to other USPs and folds into the three typical domains (finger, palm and thumb domains) (Figure 2A). The finger domain of USP18 binds a zinc ion that is co-ordinated by four cysteine residues and stabilises this domain. Without ISG15 bound, the catalytic triad of USP18 is in an inactive conformation. Two different conformations for USP18 were crystallised which differ with respect to the orientation of the finger domain. These two conformations represent an open and a closed state, i.e. states compatible and incompatible with ISG15 binding and highlight the flexibility of the finger domain (Figure 2A). Likewise, the so-called switching loop in the thumb domain of USP18 occured in an inactive or an active conformation that allows or prevents ISG15 binding, respectively.

## Binding to ISG15 and structural basis of the substrate specificity

The structure of an USP18–ISG15 complex revealed the catalytically active conformation of USP18 [35]. All three domains of USP18 contribute to binding of ISG15 (Figure 2B). In contrast with ubiquitin, ISG15 comprises two distinct Ubl domains. However, only the C-terminal Ubl domain of ISG15 interacts with USP18 whereas no interaction between the N-terminal Ubl domain and USP18 was detected and the presence of the N-terminal domain of ISG15 is dispensable for the deISGylation activity of USP18.

ISG15 comprises a characteristic hydrophobic patch in the C-terminal domain which is centred around a tryptophan residue (Trp^121^ in mouse ISG15) [16]. USP18 accomodates this hydrophobic patch of ISG15 in a shallow pocket on the surface (Figure 2C and D). This region was defined as ISG15-binding box1 (IBB-1) and is absent from ubiquitin-deconjugating USPs. Exchanging the residues in IBB-1 of USP18 with those of ubiquitin-specific USP7 rendered USP18 inactive, highlighting the importance of this pocket for the enzymatic activity. Interestingly, IBB-1 in USP18 of fish harbours more polar and hydrophilic residues. This change in surface properties is compensated by an exchange of the residues of the hydrophobic patch (tryptophan and proline in mouse ISG15) with the polar residues arginine and glutamine in fish ISG15. In contrast with mouse and human USP18, fish USP18 cannot only be modified by ISG15- but also with ubiquitin-derived activity-based probes. These probes covalently bind to the active site cysteine in an active DUB. Thus, the hydrophobic nature of IBB-1 is critical for the strict ISG15-specficity in human and mouse USP18.

## Regulation of type I interferon signalling

Beside being a highly specific isopeptidase, USP18 fulfils functions independent of its protease activity. USP18 knockout mice (USP18^−/−^) were described to develop brain injuries and show reduced life expectancy [37]. While this phenotype was first interpreted to arise from the enhanced ISGylation detected in the brain of these mice, it was shown later on that the observed phenotype persists in USP18/ISG15 double knockout mice (USP18^−/−^/ISG15^−/−^) and hence develops independent of ISG15 [38]. In contrast, USP18^C61A/C61A^ mice (129/C57BL/6 and C57BL/6 genetic backgrounds) neither show brain abnormalities nor a reduced lifespan, clearly showing that the observed phenotype in USP18^−/−^ mice does not depend on the enzymatic activity of the protease [27]. USP18^−/−^ and USP18^−/−^/ISG15^−/−^ mice are hypersensitive to LPS and polyI:C and show reduced survival after injections with these proinflammatory compounds [38]. Noteworthy, the genetic background of the transgenic animal has a major impact on the survival. Whereas USP18^−/−^ and USP18^−/−^ISG15^−/−^ mice are born at normal mendelian ratios if bred on a mixed genetic background (129, C57BL/6, Swiss Webster) [38], lack of USP18 is embryonically lethal for mice on a C57BL/6 background [39].

It was shown that USP18-deficient murine cells show a hyperactive type I interferon signalling characterised by increased and prolonged phosphorylation of the transcription factors STAT1 and STAT2 (signal transducer and activator of transcription) and enhanced expression of interferon-stimulated genes [6,40]. This also holds true for cell-specific deletions: mice that specifically lack USP18 in microglia – the resident immune cells in the brain – develop an inflammation in the white matter of the brain with hyperactivated microglia that show prolonged phosphorylation of STAT and increased expresson of type I interferon target genes [41]. This phenotype can be rescued in mice that additionally lack IFNAR1 showing that the observed phenotype is strictly dependent on the type I interferon signalling pathway [41].

In 2010, Richer et al. [42] described mice that harbour a missense mutation in USP18 in which the leucine residue at position 361 is replaced by phenylalanine. These so-called USP18-Ity9 mice were derived from a mutagenesis screen using the mutagenic agent N-Ethyl-N-Nitrosourea (ENU) that was administered to male mice on a 129S1 genetic background [42]. Similar to USP18^−/−^ mice, mice homozygous for USP18-Ity9 (USP18^Ity9/Ity9^) show perinatal lethality if bred on a C57BL/6J genetic background, whereas mice on a mixed (C57BL/6x129S1xDBA/2J), DBA/2J or 129S1 genetic background were born at normal mendelian ratios [43]. USP18^Ity9/Ity9^ mice show enhanced susceptibility to infection with *Salmonella typhimurium*, a bacterium used to study systemic *Salmonella* infections, and to infections with *Mycobacterium tuberculosis* [42,43]. Compared with wild-type mice, USP18^Ity9/Ity9^ mice only show a minimal inflammatory response early after the infection with *Salmonella* characterised by low pathogenic scores and decreased levels of IFNγ. This leads to an earlier systemic dissemination of the bacteria, increased bacterial load in several organs, a cytokine-induced septic shock and subsequent death of the infected animal [44]. USP18^Ity9/Ity9^-derived cells exhibit enhanced phosphorylation of STAT1 and increased expression of interferon-target genes after stimulation with IFNβ. Blocking the type I interferon receptor prior to infection with *Salmonella* improved survival of USP18^Ity9/Ity9^ mice. Again, USP18^Ity9/Ity9^ that additionally lack ISG15 did not differ in their susceptibility to *Salmonella* compared with USP18^Ity9/Ity9^ mice [43] showing that this phenotype is not related to the lack of deISGylation.

In contrast with USP18^−/−^ mice on mixed and C57BL/6 genetic backgrounds, USP18-deficient mice on an FVB/N genetic background do not show neurological symptoms but spontaneously develop subcutaneous leimyosarcomas [45]. However, neither growth nor survival of the formed tumours depends on the loss of USP18. Cells derived from USP18^−/−^ mice on a FVB/N genetic background did not show an altered response to IFNβ stimulation as compared with wild-type cells [45].

Repeated stimulation with IFNα causes a refractory state in which cells cannot respond to additional doses of IFNα but still respond to IFNβ [5]. This desensitisation is characterised by a reduced phosphorylation of STAT1 after a second administration of IFNα. It was shown that USP18 is responsible to induce this refractory state as USP18-deficient mice (FVB background) and cells do exhibit this kind of desensitisation after repeated injections of IFNα [5,46]. IFNα2 is used to treat patients with hepatitis C infection and the observed desensitisation after frequent injections impairs the therapeutic effect of IFNα [46]. Different infection models for hepatitis C and B virus (HCV and HBV) revealed that silencing of USP18 in hepatoma cell lines potentiates the antiviral activity of type I interferons against HBV and HCV [9,47]. In line with this observation, USP18^−/−^ mice (129 × C57BL/6 mixed background) were described to have lower levels of HBV DNA as compared with wild-type animals [48].

It was shown for glioblastoma cell lines that pretreatment of cells with IFNα sensitises the cells to TRAIL-induced apoptosis. High levels of USP18 in these cells promote resistance to this process. In line with the function of USP18 as a negative regulator of the IFN response, silencing of USP18 in gliobloastoma cells resulted in enhanced susceptibility to apoptosis after stimulation with TRAIL [49,50].

USP18-deficient mice (Sv129 × C57BL/6 mixed background) that were treated with vesicular stomatitis virus (VSV) were reported to have increased viral replication in spinal cord and brain and die within 1 week whereas wild-type mice survive the infection [51]. The enhanced susceptibility of USP18^−/−^ mice was ascribed to a defective induction of adaptive immunity. CD169^+^ macrophages in the red pulp of the spleen were described to have higher expression levels of USP18 compared with other macrophage populations. This leads to diminished type I interferon signalling and promotes virus replication in CD169^+^ macrophages which is important to induce adaptive immunity against the infection. Consequently, USP18^−/−^ mice exhibited reduced and slower T- and B-cell response against a VSV-specific antigen [51].

In 2016, Meuwissen et al. [52] described five patients from two families with autosomal recessive loss-of-function mutations in USP18 that resulted in no or abnormal transcription from the mutated allels and a complete lack of the USP18 protein. The patients developed Pseudo-TORCH syndrome that resembles a congenital infection in the absence of an infectious agent and manifests with microcephaly, enlarged ventricles and cerebral calcifications. All patients died within a few days after birth [52]. Patient-derived fibroblasts showed enhanced IFN-induced inflammation and enhanced and prolonged STAT phosphorylation which could be reversed by transduction of cells with wild-type USP18. Based on these findings, USP18 deficiency was classified as a type I interferonopathy and hence as a disease associated with up-regulated interferon signalling [52]. This highlights the critical role of USP18 in negatively regulating type I interferon signalling in humans.

## Molecular basis of USP18-mediated inhibition of type I interferon signalling

Type I interferons (in humans 12 IFNα subtypes, IFNβ, IFNε, IFNκ, IFNω) are cytokines that counteract viral infection in the innate immune response [53]. The constitutively expressed type I interferon receptor IFNAR is composed of two subunits IFNAR1 and IFNAR2. IFNAR1 and IFNAR2 are associated with the intracellular kinases and Tyrosine kinase 1 (TYK2) and JAK1 (Janus activated kinase 1), respectively. Binding of type I interferons to the cell surface induces dimerisation of the two receptor subunits and subsequent activation of the associated kinases which recruit and phosphorylate the transcription factors STAT1 and STAT2. p-STAT1 and 2 form a heterodimer that translocates into the nucleus and binds the transcription factor IRF9 (IFN-regulatory factor 9) to form the trimeric complex ISGF3 (IFN-stimulated gene factor 3). This complex binds to promoters of genes that contain an interferon-stimulated response element (ISRE) and stimulates transcription of interferon target genes (ISGs) [53,54].

USP18 is one of the smallest members of the USP family [55]. It mainly consists of the USP domain itself and does not contain additional structured domains. Thus, the inhibition of type I interferon signalling must be mediated by the catalytic domain itself. USP18 was reported to directly bind to the intracellular part of IFNAR2 and to compete with binding of JAK1 to the receptor which results in inhibition of type I interferon signalling [22]. The interface between the two proteins was mapped to a cytoplasmic region in IFNAR2 referred to as Box1-Box2, which comprises approximately 100 amino acid residues. This part of IFNAR2 was recognised by the C-terminal region of USP18 (Figure 2D) [22]. The binding of USP18 to IFNAR2 was shown to be independent of dimerisation of IFNAR2 with IFNAR1 [56]. While it was first suggested that USP18 binding to IFNAR2 does not compete with ternary complex formation between IFNAR1, IFNAR2 and IFNα2 [56]. It was reported later on that USP18 reduces binding of IFNα to the cell surface and interferes with the ability of IFNAR2 to recruit IFNAR1 [57]. This results in a loss of functional signalling complexes on the plasma membrane [57]. In 2017, Arimoto et al. [58] provided evidence that, in addition to IFNAR2, USP18 directly interacts with STAT2 and that all three proteins colocalise in cells. The DNA-binding domain as well as a coiled-coil region in the central part of STAT2 are required for binding to USP18. For USP18, both N- and C-terminal regions were described to bind to STAT2 (Figure 2D). In addition to the previously described C-terminal part of USP18 that recognises IFNAR2, an N-terminal region in USP18 was identified that contributes to the interaction with the receptor. In STAT2-deficient cells, the USP18-mediated inhibition of type I interferon signalling was reduced and the interaction between IFNAR2 and USP18 was weakened pointing to an essential role of STAT2 in USP18-mediated inhibition of the type I interferon signalling pathway [58]. In the USP18^Ity9/Ity9^ mice, the leucine at position 361 in USP18 is replaced with phenylalanine [42] and it was suggested that this mutation abolishes the interaction with IFNAR2 [41]. Residue 361 is not located on the surface of the USP18 molecule [35] and it is likely that it is not involved in direct protein–protein interactions. It was speculated that the exchange of the leucine residue with the bulkier phenylalanine might disturb the conformation of a surface loop close by which in turn interferes with binding to the interferon receptor [41]. Whether the USP18-L361F mutation also influences the catalytic activity of USP18 remains to be determined.

## Involvement of USP18 in additional intracellular pathways

Besides the well-established major functions of USP18 as a specific ISG15 isopeptidase and an inhibitor of type I interferon signalling, it was suggested that USP18 was suggested to play a role in other signalling pathways. In 2013, Liu et al. [59] reported that USP18 regulates the activation of T cells and is involved in the differentiation of T_H_17 cells. In this study, USP18 was reported to negatively regulate nuclear factor κB (NF-κB) signalling in T cells by binding to the TGFβ-activated kinase/TAK binding protein (TAB1/TAK1) complex inhibiting its deubiquitination [59]. Furthermore, USP18 was described to negatively regulate TLR-mediated NF-κB signalling: Yang et al. [60] suggested that USP18 inhibits ubiquitination of the TAK/TAB complex and the IKKα/β-NEMO (IκB kinase/NF-κB essential modulator) complex in a protease-dependent or independent manner, respectively. However, as mouse and human USP18 do not exhibit any activity towards ubiquitin, the molecular basis for this USP18-mediated inhibition of ubiquitination is unclear and might be indirect within the cellular context. In 2016, Zhang et al. [61] reported that USP18 interacts with the deubiquitinase USP20 and recruits it to stimulator of interferon genes (STING). STING is activated by cGAS (cyclic GMP–AMP synthase) which recognises dsDNA in the cytoplasm after infection with a DNA virus. USP20 was reported to deconjugate ubiquitin from STING to enhance its stability [62,63]. This suggests a role of USP18 in positively regulating expression of type I IFNs and proinflammatory cytokines after infection with a DNA virus [61].

## Open questions

It is well established that USP18 is a bifunctional protein exhibiting protease-dependent and -independent functions. While we understand well how USP18 recognises ISG15 and deconjugates it from target proteins, it is less clear how USP18 negatively regulates type I IFN signalling on the molecular level. The reported interactions between USP18 and IFNAR2 as well as STAT2 remain to be characterised biochemically and structurally. Moreover, it is so far unknown whether protease-dependent and -independent functions of USP18 influence each other and if USP18, once bound to IFNAR2 and STAT2, is still able to deISGylate target proteins. USP18^C61A/C61A^ mice are more resistant to several viral infections than wild-type mice but do not show any other obvious phenotypic abnormalities. Thus, developing an inhibitor that selectively targets USP18 catalytic activity might represent a novel pharmaceutical approach to enhance resistance to viral infections without side effects or affecting the protease-independent functions of USP18. Finally, it is conceivable that further not yet identified interaction partners of USP18 exist that modulate protease-dependent or independent functions of USP18.

## Abbreviations

DUB: deubiquitinating enzyme
HBV: hepatitis B virus
HCV: hepatitis C virus
IBB-1: ISG15-binding box1
IRF9: IFN-regulatory factor 9
ISG: interferon-stimulated gene
JAK1: Janus activated kinase 1
LPS: lipopolysaccharide
MERS: Middle East respiratory syndrome
NF-κB: nuclear factor κB
STAT: signal transducer and activator of transcription
STING: stimulator of interferon gene
TAK: transforming growth factor beta-activated kinase
TGF: transforming growth factor
TLR: toll-like receptor
TNF: tumor necrosis factor
TORCH: toxoplasmosis, other (syphilis, varicella, mumps, parvovirus and HIV), rubella, cytomegalovirus, and herpes simplex
TRAIL: TNF-related apoptosis-inducing ligand
USP: Ubiquitin-specific protease
VSV: vesicular stomatitis virus
